# Disparities in Mpox Vaccination Among Priority Populations During the 2022 Outbreak

**DOI:** 10.1093/ofid/ofad434

**Published:** 2023-08-18

**Authors:** Naseem Alavian, Ahmad Mourad, Edwin W Woodhouse, Emily Niehaus, Hayley Cunningham, Sofia Zavala, Patricia Kohler, Steven Pappas, Michael E Yarrington, Nwora Lance Okeke, Cameron R Wolfe, Gary M Cox, Kristen V Dicks, Jason E Stout

**Affiliations:** Division of Infectious Diseases, Department of Medicine, Duke University Medical Center, Durham, North Carolina, USA; Division of Infectious Diseases, Department of Medicine, Duke University Medical Center, Durham, North Carolina, USA; Division of Infectious Diseases, Department of Medicine, Duke University Medical Center, Durham, North Carolina, USA; Division of Infectious Diseases, Department of Medicine, Duke University Medical Center, Durham, North Carolina, USA; Division of Infectious Diseases, Department of Medicine, Duke University Medical Center, Durham, North Carolina, USA; Division of Infectious Diseases, Department of Medicine, Duke University Medical Center, Durham, North Carolina, USA; Division of Infectious Diseases, Department of Medicine, Duke University Medical Center, Durham, North Carolina, USA; Division of Infectious Diseases, Department of Medicine, Duke University Medical Center, Durham, North Carolina, USA; Division of Infectious Diseases, Department of Medicine, Duke University Medical Center, Durham, North Carolina, USA; Division of Infectious Diseases, Department of Medicine, Duke University Medical Center, Durham, North Carolina, USA; Division of Infectious Diseases, Department of Medicine, Duke University Medical Center, Durham, North Carolina, USA; Division of Infectious Diseases, Department of Medicine, Duke University Medical Center, Durham, North Carolina, USA; Division of Infectious Diseases, Department of Medicine, Duke University Medical Center, Durham, North Carolina, USA; Division of Infectious Diseases, Department of Medicine, Duke University Medical Center, Durham, North Carolina, USA

**Keywords:** health care disparities, mpox, vaccination, vaccine

## Abstract

**Background:**

The 2022 mpox outbreak disproportionately affected men who have sex with men and persons living with HIV (PLWH). A 2-dose mpox vaccine series was deployed in mid-2022. Structural racism and insurance status may have affected equitable vaccination.

**Methods:**

We defined 3 cohorts: PLWH with at least 1 clinic visit between 1 July 2021 and 1 July 2022 (n = 2066), HIV preexposure prophylaxis (PrEP) recipients as of 1 January 2022 (n = 262), and all mpox-vaccinated patients in our health system between 1 July 2022 and 30 November 2022 (n = 807). We identified patients with prior diagnosed sexually transmitted infections (STIs) as having a positive test result for gonorrhea, chlamydia, or syphilis between 1 July 2021–1 July 2022. The primary outcome was receipt of at least 1 dose of mpox vaccine.

**Results:**

We identified 224 (10.8%) PLWH and 50 (19.0%) PrEP patients who received at least 1 dose of mpox vaccine. Among PLWH, White race (odds ratio [OR], 1.55; 95% CI, 1.11–2.16), private insurance (OR, 1.83; 95% CI, 1.01–3.34), prior STI (OR, 3.04; 95% CI, 2.16–4.27), prior COVID-19 vaccination (OR, 3.17; 95% CI, 1.93–5.20), and prior influenza vaccination (OR, 1.42; 95% CI, 1.30–1.96) independently predicted mpox vaccination. Within the PrEP cohort, prior COVID-19 vaccination and seasonal influenza vaccination predicted mpox vaccination. Uninsured patients were vaccinated later in the outbreak than patients with private insurance (median time to vaccination, 41 days in the privately insured group vs 83 days in the uninsured group; *P* < .0001).

**Conclusions:**

Race, insurance status, prior STI, and previous receipt of other vaccines influenced uptake of mpox vaccine. Addressing health disparities and vaccine acceptance will be essential in improving future outbreak response.

## Teaser

Evaluation of our retrospective cohorts demonstrated that race, insurance status, prior sexually transmitted infection, and previous receipt of other vaccines influenced uptake of mpox vaccine. Addressing health disparities and lack of vaccine confidence will be essential in improving future outbreak response.

The 2022 mpox outbreak disproportionally affected men who have sex with men, persons living with HIV (PLWH), and those with recently acquired sexually transmitted infections (STIs) [1–3]. The US Department of Health and Human Services deployed a free 2-dose JYNNEOS vaccine series (Modified Vaccinia Ankara; Bavarian Nordic) in June 2022 to limit infection and morbidity [4, 5]. Given the recent racial and ethnic disparities in patient outcomes and vaccine uptake during the COVID-19 pandemic, health agencies were encouraged to measure health care disparities and embed equity strategies as a core aspect of mpox vaccination efforts [6]. Current data suggest that while Black and Hispanic individuals made up approximately two-thirds of mpox cases, they were underrepresented among vaccinated individuals in the United States [7–9]. Among persons who received ≥1 dose of the JYNNEOS vaccine, 51.4% were non-Hispanic White, 22.5% Hispanic, and only 12.6% Black [10].

While some studies have explored the impact of race and ethnicity on vaccination [7–11], data are lacking on how other disparities, such as socioeconomic stability, insurance status, or other social determinants of health, are independently related to vaccine uptake. Additionally, data are lacking in characterizing the correlation between the likelihood of acquiring mpox and the probability of being vaccinated. This may suggest ongoing relevance of Tutor Hart's inverse care law, in which availability of health care—in this case, vaccination—varies inversely with the need for it in the population served [12]. Better understanding of the independent association of these factors on vaccination may inform public policy and interventions to improve vaccine equity.

Our study describes the characteristics of patients vaccinated and examines correlates of mpox vaccine status in a single health system in the southeastern United States, an area with well-characterized HIV-related health disparities [13, 14]. This work complements published national data regarding mpox vaccination by providing detailed information on vaccine uptake at a local level within priority populations.

## METHODS

### Patient Consent Statement

The Duke University institutional review board approved this study before it began, and informed consent was not required or obtained, in accordance with title 45 CFR §46.

### Study Setting and Cohort

We used the Duke electronic medical record interface to retrospectively identify and define 3 cohorts of patients based on varying perceived susceptibility to mpox infection as well as degree of engagement in care [15]:

Cohort 1: PLWH with at least 1 HIV clinic visit at our institution between 1 July 2021 and 30 November 2022
Cohort 2: patients receiving HIV preexposure prophylaxis (PrEP) with an active prescription for emtricitabine-tenofovir disoproxil fumarate or emtricitabine-tenofovir alafenamide fumarate as of 1 January 2022
Cohort 3: partially overlapping cohorts 1 and 2, all patients who received at least 1 dose of mpox vaccine at a site within the Duke University Healthcare System (DUHS) between 1 July 2021 and 30 November 2022

The DUHS offered mpox vaccination at the following sites: the DUHS Adult Infectious Disease Clinic, 2 outpatient clinics that provide laboratory and vaccine services, a pop-up clinic at a Durham Pride Parade event on 24 September 2022, and a pop-up clinic at an event sponsored by the Latinx Advocacy Team & Interdisciplinary Network for COVID-19.

### Patient Characteristics and Definitions of Variables

Demographic data collected for this project included age, sex at birth, self-reported race, self-reported ethnicity, area of residence, and insurance status as of 1 July 2022. We collected clinical information, including HIV status and recent STI. Recent STI was defined by a positive result from a gonorrhea or chlamydia nucleic acid amplification test from any site (urine, rectal, or oropharyngeal) and/or a rapid plasma reagin during the year prior to onset of the outbreak in the United States (1 July 2021–30 June 2022). Insurance status was ascertained for each patient as of 1 July 2022. As a surrogate for individual vaccine acceptance or hesitancy, prior COVID-19 and influenza vaccination status was also collected. This was defined as receipt of at least 1 vaccine (any manufacturer) for COVID-19 prior to 1 July 2022 and receipt of influenza vaccine in the preceding influenza season (1 August 2021–31 March 2022). Mpox vaccination was defined by receipt of at least 1 dose of the JYNNEOS vaccine at a DUHS site. We used the Social Vulnerability Index (SVI) from the Centers for Disease Control and Prevention (CDC) and the Agency for Toxic Substances and Disease Registry to categorize social vulnerability based on individual residential address. This index uses US Census data to determine the social vulnerability of every census tract based on 16 social factors [16]. The SVI ranges from 0 to 1, with a higher score indicating higher social vulnerability.

### Statistical Analysis

The primary outcome variable for this study was receipt of at least 1 dose of JYNNEOS mpox vaccine. Specific predictors of mpox vaccination included demographics (age, sex at birth, race, ethnicity), insurance status, SVI, new STI within the past year, and prior COVID-19 vaccination and seasonal influenza vaccination (surrogate for individual vaccine acceptance or hesitancy). Categorical data were evaluated as proportions with the χ^2^ test, and continuous data were compared with unpaired *t* tests or the Kruskal-Wallis test, as appropriate for the distributions and number of groups compared, with the Dunn test used to determine pairwise significance if an overall Kruskal-Wallis test result was significant. Statistical significance was set at α < .05. Factors significantly associated with mpox vaccination in univariate analysis were entered into a multivariable unconditional logistic regression model. Sex was not included in the multivariable model because the number of vaccinated females within our cohorts was low. R version 4.0.0 (R Core Team) with the RStudio interface was used for data analysis.

## RESULTS

### HIV Clinic Patient Cohort

We identified 2066 unique PLWH who had a clinic or laboratory visit at the Duke University Adult Infectious Disease clinic between 1 July 2021 and 30 November 2022 (Table 1). Among this cohort, 1234 (59.7%) were Black; 1524 (73.8%) were male; 102 (4.9%) were Hispanic ethnicity; and 276 (13.4%) had a history of ≥1 recent STI (syphilis, chlamydia, or gonorrhea). Insurance coverage included 1226 (59.3%) patients with private insurance, 265 (12.8%) Medicare, 219 (10.6%) Medicaid, and 304 (14.7%) uninsured.

**Table 1.** Characteristics of Patients Living With HIV Stratified by Mpox Vaccination Status

	Patients, No. (%)			
Characteristic	Total (n = 2066)	No Vaccination (n = 1842)	Vaccination (n = 224)	*P* Value
Sex				<.001
Male	1524 (73.8)	1301 (70.6)	223 (99.6)	
Female	539 (26.0)	538 (29.2)	1 (0.4)	
Unknown	3 (0.2)	3 (0.2)	…	
Age, y, mean ± SD	…	50.50 ± 13.76	45.85 ± 12.30	<.001
Ethnicity				.123
Non-Hispanic	1836 (88.9)	1628 (88.4)	208 (92.9)	
Hispanic	102 (4.9)	94 (5.1)	8 (3.6)	
Unknown	128 (6.2%)	120 (6.5)	8 (3.6)	
Race				.070
Black or African American	1234 (59.7)	1113 (60.4)	121 (54.0)	
Caucasian/White	610 (29.5)	529 (28.7)	81 (36.2)	
Other	222 (10.7)	200 (10.9)	22 (9.8)	
Insurance type^a^				<.001
Private	1236 (56.0)	1076 (59.4)	160 (72.1)	
Uninsured	306 (14.8)	273 (15.1)	33 (14.9)	
Medicaid	224 (10.8)	210 (11.6)	14 (6.3)	
Medicare	266 (10.9)	251 (13.9)	15 (6.8)	
STI in the year				
Preceding or during the outbreak	344 (16.7)	247 (13.4)	97 (43.3)	<.001
Preceding the outbreak	276 (13.3)	205 (11.1)	71 (31.7)	<.001
Vaccination				
COVID-19^b^	1563 (75.7)	1366 (76.4)	197 (90.0)	<.001
Seasonal influenza^c^	1105 (53.5)	962 (52.2)	143 (63.8)	.001

Abbreviation: STI, sexually transmitted infection.

a Insurance data were missing in 50 patients (2.4%).

b Receipt of at least 1 vaccine (any manufacturer) for COVID-19 prior to 1 July 2022.

c Receipt of influenza vaccine in the preceding influenza season 1 August 2021–31 March 2022.

**Figure 1.**
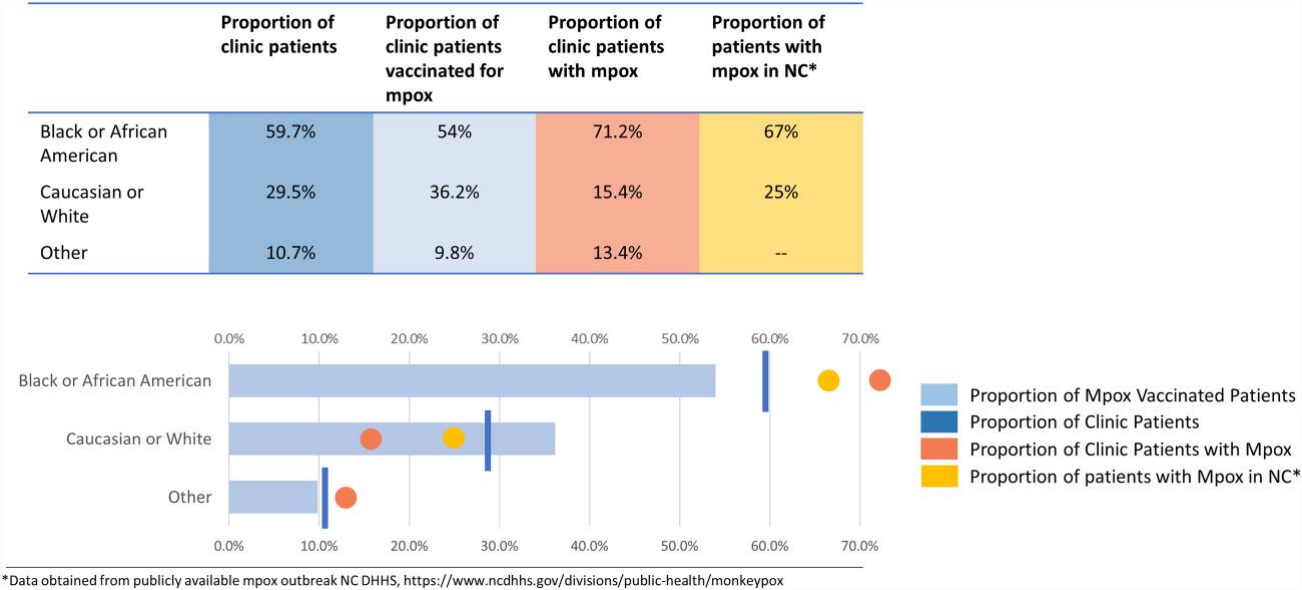
Proportion of persons living with HIV vaccinated for mpox in relation to HIV clinic composition and mpox outbreak in North Carolina.

Overall, 224 (10.8%) PLWH in our clinic received at least 1 dose of the mpox vaccine. The proportion of Black patients vaccinated (121/224, 54%) was lower than the overall proportion of Black clinic patients (1234/2066, 59.7%), whereas the proportion of White patients vaccinated (81/224, 36.2%) was greater than the proportion of White clinic patients (610/2066, 29.5%; Figure 1). The proportion of Black patients vaccinated was also lower than the proportion of Black patients diagnosed with mpox in the clinic population and in all of North Carolina [17]. Conversely, the proportion of White patients vaccinated was greater than the proportion of White patients with mpox in the clinic population and in all of North Carolina (Figure 1).

**Figure 2.**
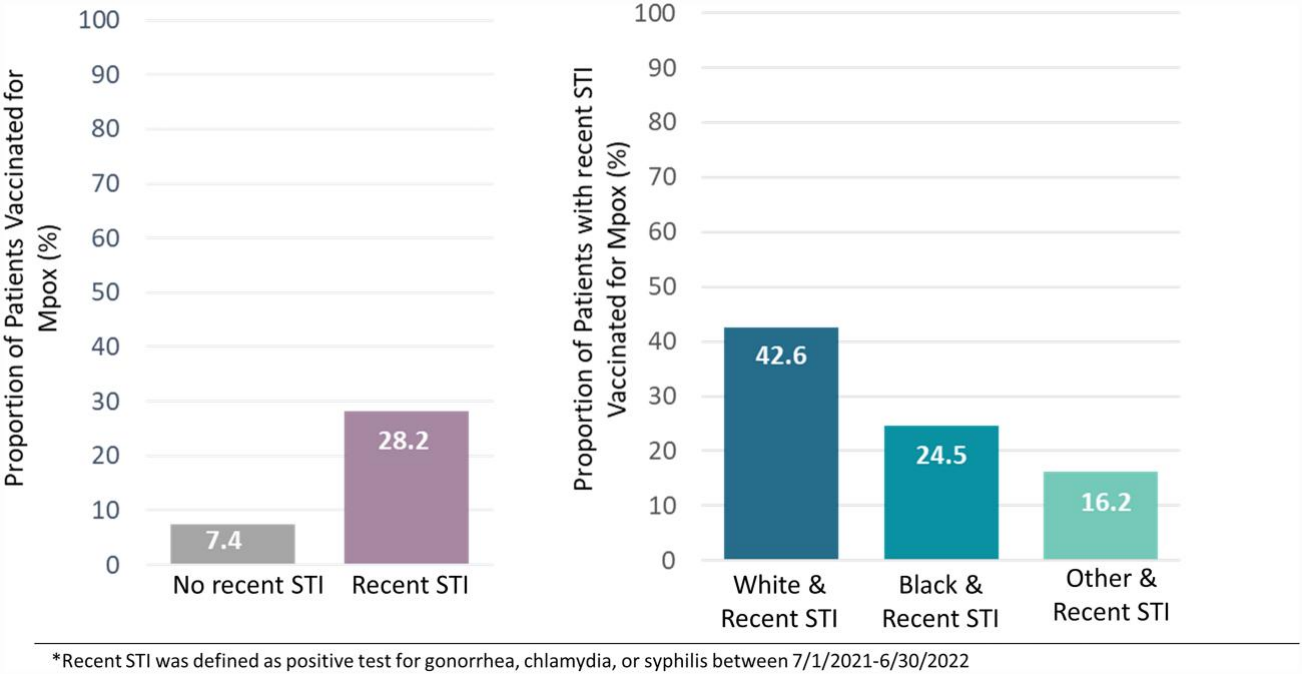
Proportion of persons living with HIV vaccinated for mpox based on identification of a recent sexually transmitted infection.

In multivariable analysis (Table 2), White persons had greater odds of vaccination than Black persons (odds ratio [OR], 1.47; 95% CI, 1.07–2.02). Patients with private insurance had higher odds of mpox vaccination as compared with patients with Medicaid (OR, 2.03; 95% CI, 1.07–2.02). Prior vaccination for COVID-19 (OR, 3.17; 95% CI, 1.93–5.20) or influenza (OR, 1.42; 95% CI, 1.03–1.96) was associated with higher odds of receiving the mpox vaccine. Recent STI diagnosis was associated with greater odds of mpox vaccination (OR, 4.86; 95% CI, 3.59–6.59) vs no recent STI; however, an overall small proportion of patients with recent STI was vaccinated. Only 28.2% (97/344) of patients with prior STI received vaccination. The missed opportunity to vaccinate patients with a recent STI was more pronounced for non-White patients as compared with White patients (Figure 2).

**Table 2.** Odds Ratio of Mpox Vaccination Among Persons Living With HIV Engaged in the HIV Clinic

Term	Mpox Vaccinated (n = 224)	Odds Ratio (95% CI)	*P* Value
Age	…	0.97 (.03–.18)	<.001
Race			
Black	121	1 [Reference]	…
White	81	1.55 (1.11–2.16)	.009
Other	22	0.83 (.49–1.39)	.694
Insurance class			
Medicaid	14	1 [Reference]	…
Private	160	1.83 (1.01–3.34)	.048
Uninsured	33	1.47 (.75–2.90)	.266
Medicare	15	1.11 (.50–2.48)	.803
Recent STI diagnosis	71	3.04 (2.16–4.27)	<.001
Vaccinated for COVID-19	197	3.17 (1.93–5.20)	<.001
Influenza (2021–2022 season)	143	1.42 (1.03–1.96)	.031

Abbreviation: STI, sexually transmitted infection.

### Cohort of Patients Receiving PrEP

We identified 262 patients with a recent visit for PrEP and an active prescription for oral PrEP as of January 2022 (Table 3). This cohort was 54.2% White, 29.6% Black, and 14.5% Hispanic. Most patients receiving PrEP were uninsured (66%); among them, 50 (19%) received the mpox vaccine in our health system. There were no significant differences in race, ethnicity, or insurance status between unvaccinated and vaccinated patients receiving PrEP. Those who were vaccinated for mpox were more likely to have received prior vaccination for COVID-19 (*P* = .008) and influenza (*P* = .020). In multivariable analysis prior vaccinations for COVID-19 (OR, 4.84; 95% CI, 1.03–22.8) and influenza (OR, 1.51; 95% CI, .72–3.2) were associated with higher odds of mpox vaccination, although the association with influenza vaccination did not reach statistical significance.

**Table 3.** Characteristics of Patients Receiving Preexposure Prophylaxis Based on Mpox Vaccination Status

	Patients, No. (%)			
Characteristic	Total (n = 262)	No Vaccination (n = 212)	Vaccination (n = 50)	*P* Value
Sex: male	242 (92.3)	193 (91.0)	49 (98.0)	<.001
Age, y, mean ± SD	…	38.25 ± 12.9	40.15 ± 11.9	.068
Ethnicity				.807
Non-Hispanic	177 (67.6)	171 (80)	41 (82)	
Hispanic	38 (14.5)	32 (15.1)	6 (12)	
Unknown/declined	12 (4.6)	9 (4.2)	3 (6)	
Race				.070
Black or African American	75 (29.6)	63 (29.7)	12 (24.0)	
Caucasian/White	142 (54.2)	112 (52.8)	30 (60.0)	
Asian	8 (3.1)	8 (3.8)	0 (0)	
American Indian or Alaskan Native	5 (1.9)	4 (1.9)	1 (2.0)	
Other	13 (5)	11 (5.2)	2 (4.0)	
Not reported/declined	18 (6.9)	13 (6.1)	5 (10.0)	
Insurance type				.816
Private	81 (30.9)	64 (30.2)	17 (34.0)	
Uninsured	173 (66)	141 (66.5)	32 (64.0)	
Medicaid	3(1.1)	3 (1.4)	0 (0.0)	
Medicare	5(1.9)	4 (1.9)	1 (2.0)	
COVID-19 vaccination	215 (82.1)	167 (78.8)	48 (96.0)	.008
Seasonal influenza vaccination	153 (58.4)	116 (54.7)	37 (74.0)	.020

### Cohort of All Patients Who Received Mpox Vaccination Within the DUHS

**Figure 3.**
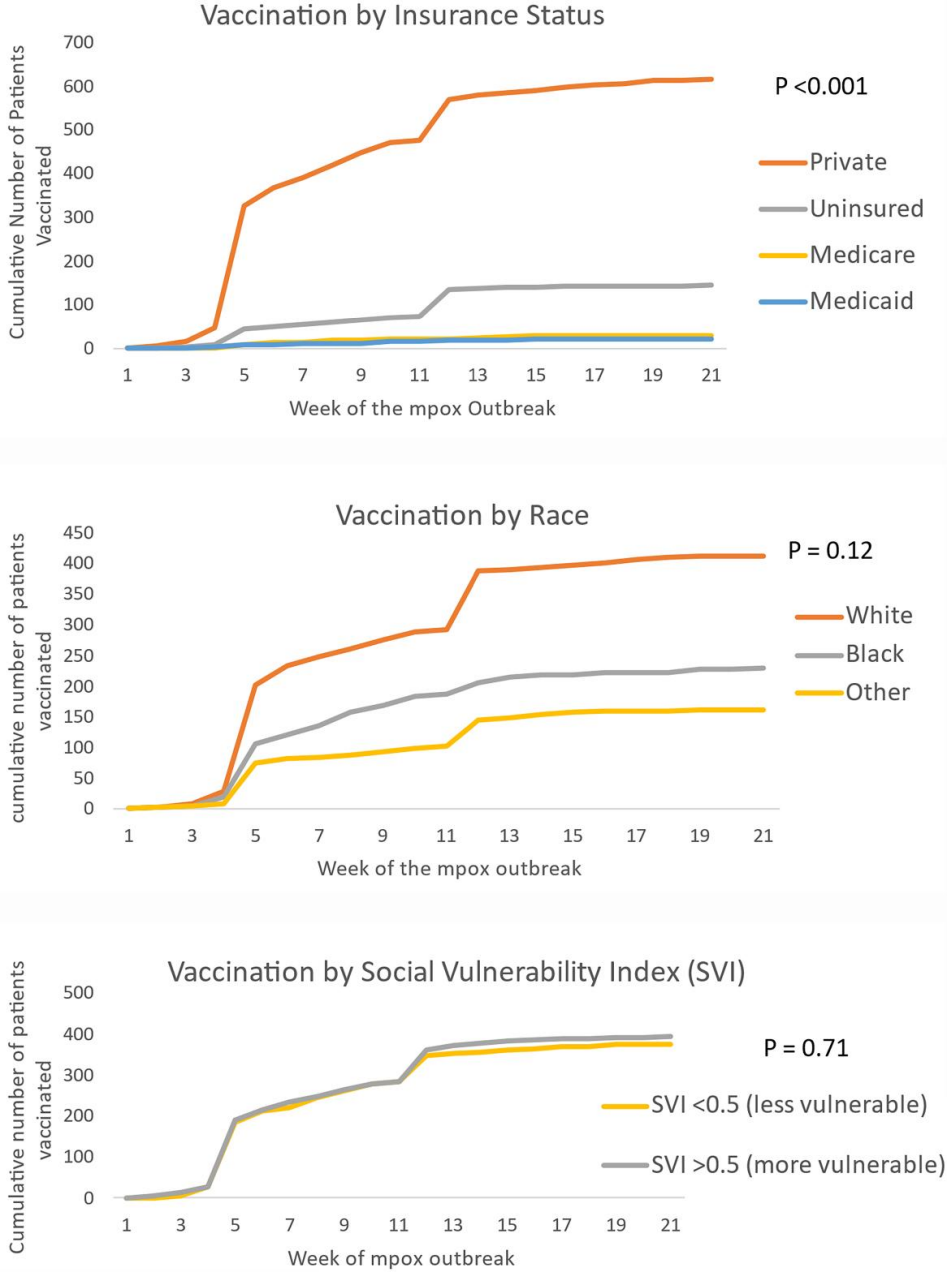
Time to mpox vaccination for all patients by week, starting 7/1/2022.

Overall, within our health system, 807 patients received at least 1 dose of mpox vaccine (87.5% male, 51.4% White, 28.6% Black). Of these patients, 223 (28.9%) were PLWH who were followed in our health system's HIV clinic, and 59 (7.3%) were patients receiving PrEP within the DUHS. The majority had private insurance (76.1%) and only 17.8% were uninsured. The 2-dose series was completed in 505 patients (62.5%; Table 4). Vaccination for patients with private insurance occurred earlier in the outbreak as compared with other types of insurance (Figure 3). The median time of vaccine administration was 41.0 days for privately insured vs 55.5 days for Medicare, 59.0 days for Medicaid, and 83.0 days for uninsured (*P* < .001 for overall difference by Kruskal-Wallis test and for the difference between uninsured and privately insured patients by Dunn test; comparisons among other insurance groups were not significant). Although more White patients were vaccinated for mpox overall, time to vaccination did not differ by race (*P* = .12) or if the individual lived in a location with a higher SVI (*P* = .71; Figure 3).

**Table 4.** Characteristics of Patients Vaccinated for Mpox Within a Single Health System (n = 807)

Characteristic	No. (%)
Sex	
Male	705 (87.5)
Female	96 (12.0)
Unknown	1 (0.1)
Age, y, mean ± SD	38.1 ± 17.6
Race	
Black or African American	229 (28.6)
Caucasian/White	412 (51.4)
Asian	41 (5.1)
American Indian or Alaskan Native	10 (1.2)
Other	17 (9.8)
Not reported/declined	85 (10.5)
Ethnicity	
Non-Hispanic	676 (84.3)
Hispanic	52 (6.5)
Unknown/not reported	74 (9.2)
Insurance type	
Private	614 (76.1)
Uninsured	144 (17.8)
Medicaid	21 (2.6)
Medicare	28 (3.4)
No. of vaccine doses received	
1	302 (37.4)
2	505 (62.5)
Patient group	
HIV positive	233 (28.9)
Preexposure prophylaxis	59 (7.3)
Other^a^	515 (63.8)

a Unknown HIV status or HIV negative.

## DISCUSSION

In our cohort study evaluating predictors of mpox vaccination in a single health care system during the 2022 mpox outbreak, we identified 3 key findings: (1) rates of mpox vaccination were low among groups with a higher likelihood of mpox acquisition; (2) White patients and patients with private insurance had higher odds of receiving at least 1 dose of mpox vaccine; and (3) prior vaccine acceptance (defined by uptake of COVID-19 or influenza vaccine) was associated with mpox vaccine uptake.

Overall, rates of mpox vaccination were low among PLWH and patients receiving PrEP in our health system. Among those with an STI in the year prior to the mpox outbreak, less than one-third received at least 1 dose of mpox vaccine. Vaccination among individuals with a higher likelihood of mpox acquisition based on the CDC's definition was similarly low at a national level, with only 23% fully vaccinated for mpox [18]. Identifying barriers to vaccination is crucial to achieve higher vaccination rates and reduce morbidity; such barriers are not only relevant to the mpox outbreak but more broadly to engagement in care of individuals with a higher likelihood of acquisition of HIV and STIs.

We found that private insurance was associated with higher odds of receiving at least 1 dose of mpox vaccine in our HIV clinic population. The mpox vaccine was provided at no cost. Therefore, this association with insurance status is unlikely to indicate a direct relationship between health insurance and the ability to receive the mpox vaccine. More likely, health insurance was a surrogate marker for socioeconomic status and other factors that could influence vaccination. Indirect barriers associated with low income, such as difficulty getting time off from work, transportation, and health literacy, may have mediated the relationship between insurance status and mpox vaccination.

Race was an independent predictor of mpox vaccination in our HIV clinic cohort. While a majority of patients diagnosed with mpox in our health system (71%) and in North Carolina (67%) were Black [17], the majority of patients (51.4%) vaccinated for mpox in our health system were White. This finding is consistent with disparities reported in recent reviews of vaccination demographics, in which White persons accounted for a greater proportion of vaccinated individuals as compared with non-White persons [7, 11]. Of the 15 028 persons vaccinated for mpox in North Carolina, the Department of Health and Human Services reported that 60.76% identified as White, 27.3% Black/African American, and 9.85% Hispanic/Latino [19]. A recent nationwide report describing mpox vaccination coverage among persons at risk for mpox revealed that approximately 1 in 8 first-dose recipients were Black, although this group accounted for an estimated 1 in 3 mpox cases [18]. The disparities that we found locally among our patients are in line with the disparity at the national level, although not as drastic. The CDC reported that nearly 4-fold more White non-Hispanic individuals were fully vaccinated as compared with those who were Black non-Hispanic [7].

Many factors could explain the racial disparities seen in this study. The impact of structural racism and systemic barriers on vaccine uptake has been described in COVID-19 and influenza vaccination efforts [20–22]. Studies have documented an association between experiences of racial discrimination and vaccine acceptance or hesitancy [23–25]. Racial discrimination is linked to feelings of racial fairness and racial consciousness and may have an impact on trust, knowledge, and attitudes surrounding vaccination. Additionally, barriers such as stigma and privacy concerns have been reported as affecting mpox vaccine acceptability and uptake [26]. Stigma associated with heath care engagement and seeking medical treatments plays a role in racial and ethnic HIV disparities [27, 28] and racial disparities in health engagement for LGBTQ individuals (lesbian, gay, bisexual, transgender, and queer) [20, 29]. Obstacles related to access, such as distance to vaccine sites, lack of transportation, and inflexible work hours, may hinder vaccine access in vulnerable communities [30]. In our health system, vaccination mostly took place at a clinic on the hospital campus; transportation to this site and the need to pay for parking may have reduced vaccine uptake. Our largest outreach effort included a pop-up clinic at a Durham pride event, which appears to have accounted for a spike in vaccination late in September 2022. Our data suggest that those who received vaccination at the time of the event were predominantly White with health insurance. Innovative approaches will be necessary to effectively reach populations most affected by mpox who experienced mpox vaccination disparities, such as Black/African American and Hispanic/Latino gay, bisexual, and other men who have sex with men, as well as people who are transgender.

In contrast to PLWH engaged in our clinic, we found no differences in vaccination rates by race, ethnicity, or insurance status among patients receiving PrEP. This finding may be due to uncaptured sociodemographic characteristics and a differential in baseline likelihood of mpox acquisition. Approximately two-thirds of our PrEP patients were uninsured, and half identified as White. Patients receiving PrEP likely interact with the health care system differently (ie, seeking preventive care). Interventions to improve access and opportunity for vaccination should be uniquely tailored to this group.

In our study, prior COVID-19 and seasonal influenza vaccination were independent predictors of vaccination in PLWH and the PrEP cohort. This is consistent with studies in the US general population showing that attitudes about COVID-19 vaccination correlate with general vaccine acceptability or hesitancy [31]. COVID-19 vaccination and influenza vaccination were independent and additive predictors of mpox vaccination, which suggests a “dose-response” behavioral effect of vaccine acceptance. Furthermore, prior vaccination is likely a surrogate for engagement in care and trust in provider recommendations [31, 32].

Operational barriers may have affected vaccination efforts. In our health system, uncertainty in vaccine quantity and delivery limited efforts to plan and publicize outreach vaccine campaigns. In some cities, the CDC Mpox Vaccine Equity Pilot Program supported innovative vaccination efforts. Reports from this program suggest that specific funding, vaccine allocation assurances, and strategic support aimed at reaching populations affected by vaccine disparities may mitigate operational and structural barriers in vaccine distribution [6]. Characterizing the success of such pilot programs and developing means to scale such efforts may improve broader vaccine distribution and uptake in future outbreaks or public health emergencies.

While our study is unique in its characterization of mpox vaccination among groups of varying likelihoods of mpox acquisition, it does have limitations. Vaccination status in this study was defined by vaccine receipt within our health system and did not account for patients who were vaccinated at other sites, such as the county health department. Our health system was, however, responsible for providing over one-third (37.5%) of all JYNNEOS doses delivered in the surrounding county [19]. A large proportion of our cohort had missing data on sexual orientation, identity, and practices, so we could not include these important elements in the analysis. Additionally, we were unable to capture prior STIs diagnosed outside our health system. Regarding limitations with how we aggregated racial/ethnic groups, we collapsed non-White and non-Black individuals into a single group, given their low numbers, thereby limiting our characterization of vaccine correlates among some racial and ethnic groups. Last, insurance status was used as a proxy for socioeconomic status, although insurance is an imperfect proxy that does not capture the myriad social and economic factors that affect health outcomes. Use of insurance status in analysis provides an interesting view, however, into how income level affects access to or uptake of new therapies, testing, or vaccination in the setting of new or emerging infections.

Our study provides insight into the mpox vaccine response of a single health care system during the 2022 mpox outbreak. We found that despite intent for equitable vaccine distribution and efforts to provide community outreach, significant racial and socioeconomic disparities in vaccination occurred. Further research of interventions to reach populations with higher likelihood of mpox acquisition and those most likely to experience health care disparities will be essential in mitigating health care inequity.

## Notes

***Author contributions.*** Conception and design: all authors. Data collection: A. M., M. E. Y., J. E. S. Analysis and interpretation of results: all authors. Writing–original draft: N. A. Writing–review and editing: J. E. S., A. M., K. V. D., E. N.., C. R. W. All authors reviewed the results and approved the final version of the manuscript.

***Financial support*.** This project was funded by the Duke Center for AIDS Research, National Institutes of Health grant 5P30 AI064518. A. M. and J. E. S. were the recipients of the grant.
